# Clinical Behavior, Mutational Profile and T-Cell Repertoire of High-Grade Neuroendocrine Tumors of the Head and Neck

**DOI:** 10.3390/cancers15092431

**Published:** 2023-04-24

**Authors:** Santiago Cabezas-Camarero, Vanesa García-Barberán, Javier David Benítez-Fuentes, Miguel J. Sotelo, José Carlos Plaza, Alejandro Encinas-Bascones, Óscar De-la-Sen, Farzin Falahat, Jesús Gimeno-Hernández, Manuel Gómez-Serrano, Fernando Puebla-Díaz, Manuel De-Pedro-Marina, Maricruz Iglesias-Moreno, Pedro Pérez-Segura

**Affiliations:** 1 Medical Oncology Department, Hospital Clínico Universitario San Carlos, Instituto de Investigación Sanitaria San Carlos (IdISSC), 28040 Madrid, Spain; javier.fuentes@wales.nhs.uk (J.D.B.-F.); pedro.perez@salud.madrid.org (P.P.-S.); 2 Molecular Oncology Laboratory, Hospital Clínico Universitario San Carlos, Instituto de Investigación Sanitaria San Carlos (IdISSC), 28040 Madrid, Spain; vanesagbes2@yahoo.es; 3 Medical Oncology Department, Aliada Cancer Center, Lima 15036, Peru; 4 Medical Oncology Department, Clínica San Felipe, Lima 15072, Peru; 5 Medical Oncology Department, Hospital María Auxiliadora, Lima 15801, Peru; 6 Pathology Department, Hospital Clínico Universitario San Carlos, 28040 Madrid, Spain; 7 Maxillofacial Surgery Department, Hospital Clínico Universitario San Carlos, 28040 Madrid, Spain; 8 Otolaryngology-Head and Neck Surgery Department, Hospital Clínico Universitario San Carlos, 28040 Madrid, Spain; 9 Radiation Oncology Department, Hospital Clínico Universitario San Carlos, 28040 Madrid, Spain

**Keywords:** head and neck cancer, neuroendocrine, T-cell repertoire, tumor mutational burden, immunotherapy

## Abstract

**Simple Summary:**

Among 1083 patients with HN cancer, 11 patients with neuroendocrine carcinomas (NECs) of the head and neck (HN) were identified. In our series, HN NECs diagnosed with localized or locally advanced disease achieved long-lasting survival with multimodality treatment. HN NECs harbored mutations in TP53, HFN1A and RB1, among others, had a median TMB of 6.72 muts/Mb and showed wide TCR repertoires. Among three patients with metastatic disease that received anti-PD1 therapy, there were two long-lasting responders.

**Abstract:**

Neuroendocrine carcinomas (NECs) of the head and neck (HN) account for <1% of HN cancers (HNCs), with a 5-year overall survival (OS) <20%. This is a retrospective study of HN NECs diagnosed at our institution between 2005 and 2022. Immunohistochemistry and next-generation sequencing (NGS) were used to evaluate neuroendocrine markers, tumor mutational burden (TMB), mutational profiles and T-cell receptor repertoires. Eleven patients with high-grade HN NECs were identified (male:female ratio 6:5; median age 61 (Min–Max: 31–86)): nasoethmoidal (3), parotid gland (3), submaxillary gland (1), larynx (3) and base of tongue (1). Among *n* = 8 stage II/IVA/B, all received (chemo)radiotherapy with/without prior surgery or induction chemotherapy, with complete response in 7/8 (87.5%). Among *n* = 6 recurrent/metastatic patients, three received anti-PD1 (nivolumab (2), pembrolizumab (1)): two achieved partial responses lasting 24 and 10 months. After a median follow-up of 30 and 23.5 months since diagnosis and since recurrent/metastatic, median OS was not reached. Median TMB (*n* = 7) was 6.72 Mut/Mb. The most common pathogenic variants were TP53, HNF1A, SMARCB1, CDKN2A, PIK3CA, RB1 and MYC. There were 224 median TCR clones (*n* = 5 pts). In one patient, TCR clones increased from 59 to 1446 after nivolumab. HN NECs may achieve long-lasting survival with multimodality treatment. They harbor moderate-high TMBs and large TCR repertoires, which may explain responses to anti-PD1 agents in two patients and justify the study of immunotherapy in this disease.

## 1. Introduction

Neuroendocrine tumors (NETs) encompass different types of tumors with different histopathologic characteristics, biological aggressiveness and thereby different clinical behaviors [1,2]. Among NETs, several subtypes are recognized: carcinoid tumors, atypical carcinoids and neuroendocrine carcinomas (NECs), the latter including large-cell and small-cell NECs. NETs and NECs may develop in almost any organ or region throughout the body. They are usually diagnosed within the gastroenteropancreatic area (so-called GEP-NETs) and in the lungs, where the most common are small-cell lung cancer (SCLC) and lung carcinoids [1,2,3]. However, other areas are well-known to develop NETs, although with a lower frequency, such as the head and neck region or the urogenital tract [1,3,4,5,6].

NETs of the HN region, and in particular NECs, are very rare disease entities, with the latter accounting for less than 1% of HN cancers, with an estimated 5-year overall survival (OS) below 20% [3,4,7]. Histologically, they are similar to NECs developing from non-HN areas. These tumors harbor cells of small-to-intermediate size and necrosis, have high proliferative indexes and show positive staining for one or more neuroendocrine markers such as synaptophysin, chromogranin and CD56 [3]. They are aggressive malignancies, closely linked to tobacco smoking, and most commonly develop in the larynx, sinonasal area and salivary glands [3]. Laryngeal, salivary gland and sinonasal NECs account for <0.5%, <1% and 1% of malignancies within these subsites. Very limited evidence exists on the mutational profile and T-cell repertoire of these tumors. Our aim was to retrospectively review the cases of NECs of the HN region diagnosed at our institution in the past eighteen years and describe their pathological characteristics, molecular and immune profiles and their clinical behavior and therapeutic management.

## 2. Materials and Methods

### 2.1. Patients

Retrospective study of HN NECs at Hospital Clinico Universitario San Carlos (Madrid, Spain) since 2005. The Institutional Review Board of Hospital Clínico Universitario San Carlos approved this study in accordance with the principles outlined in the ‘World Medical Association Declaration of Helsinki’. A signed informed consent form was obtained from all the patients prior to study participation. Informed consent was waived in case of deceased patients. The REporting recommendations for tumour MARKer prognostic studies (REMARK) guidelines were followed to conduct this study [8].

Overall response rate (ORR) was evaluated according to RECIST 1.1 [9]. Overall survival (OS) since initial diagnosis (IDx), progression-free survival (PFS) and OS since the recurrent/metastatic (R/M) disease and since the start of anti-PD1 agents were defined as the time from IDx until death from any cause and the time from first-line therapy or since the start of anti-PD1 therapy until progression or death from any cause, respectively.

### 2.2. Immunohistochemistry Studies

Antibodies (all from Dako North America, Carpintería, CA, USA) against cytokeratins (CKs) AE1/AE3 (clones AE1/AE3; dilution 1:50) and CK 5/6 (clone D5/16 B4; dilution 1:50) and against chromogranin (clone DAK-A3; dilution 1:100), synaptophysin (clone DAK-SYNAP; dilution 1:200), CD56 (clone 123C3; dilution 1:50), CD99 (clone 12E7; dilution 1:1000), neuronal specific enolase (clone BBS/NC/VI-H14; dilution 1:200), Ki67 (clone MIB-1; dilution 1:100) and PD-L1 (clone 22C3; dilution 1:50) were used for the characterization of each of the tumors through immunohistochemistry studies. Expression of PD-L1 was considered positive when there was ≥1% of tumor cells with membranous staining [10].

### 2.3. Tumor Mutational Load, Mutational Profile and T-Cell Receptor Repertoire Studies

Four to eight sections of paraffin-embedded tumor tissue, with the tumor region selected by a pathologist, were obtained, and DNA extraction was performed using the ‘QIAamp DNA FFPE Kit GenRead’ kit (QIAGEN, Germantown, MD, USA). The QUBIT 3.0 fluorometer instrument (Thermo Fisher Scientific, Waltham, MA, USA) was used for DNA quantification.

The Oncomine Tumor Mutational Load Assay (Thermo Fisher Scientific, Waltham, MA, USA) was used for next-generation sequencing (NGS) tumor mutational burden (TMB) and mutational profile analyses. Oncomine TCR-Beta-SR assay (Thermo Fisher Scientific, Waltham, MA, USA) was used for NGS analysis of the nucleotide sequence of the CDR3 region coding for the T-cell receptor beta (TCRβ) chain. Libraries were loaded on an Ion 540 chip using the Ion Chef Instrument, sequenced in an Ion GeneStudio S5 System (Thermo Fisher Scientific, Waltham, MA, USA) and analyzed using the Ion Reporter version 5.12 (Thermo Fisher Scientific, Waltham, MA, USA). Shannon’s diversity and evenness were defined and calculated as previously described [11].

### 2.4. Statistical Analysis

A descriptive analysis of demographic and clinicopathological data was conducted. PFS and OS were estimated using the Kaplan–Meier method. Pearson’s and Spearman’s correlation tests were used to evaluate parametric and non-parametric correlations, respectively. SPSS Statistics for MacOS version 23.0 (Armonk, NY, USA) was used for all statistical analyses.

## 3. Results

### 3.1. Baseline Characteristics

**Figure 1:**
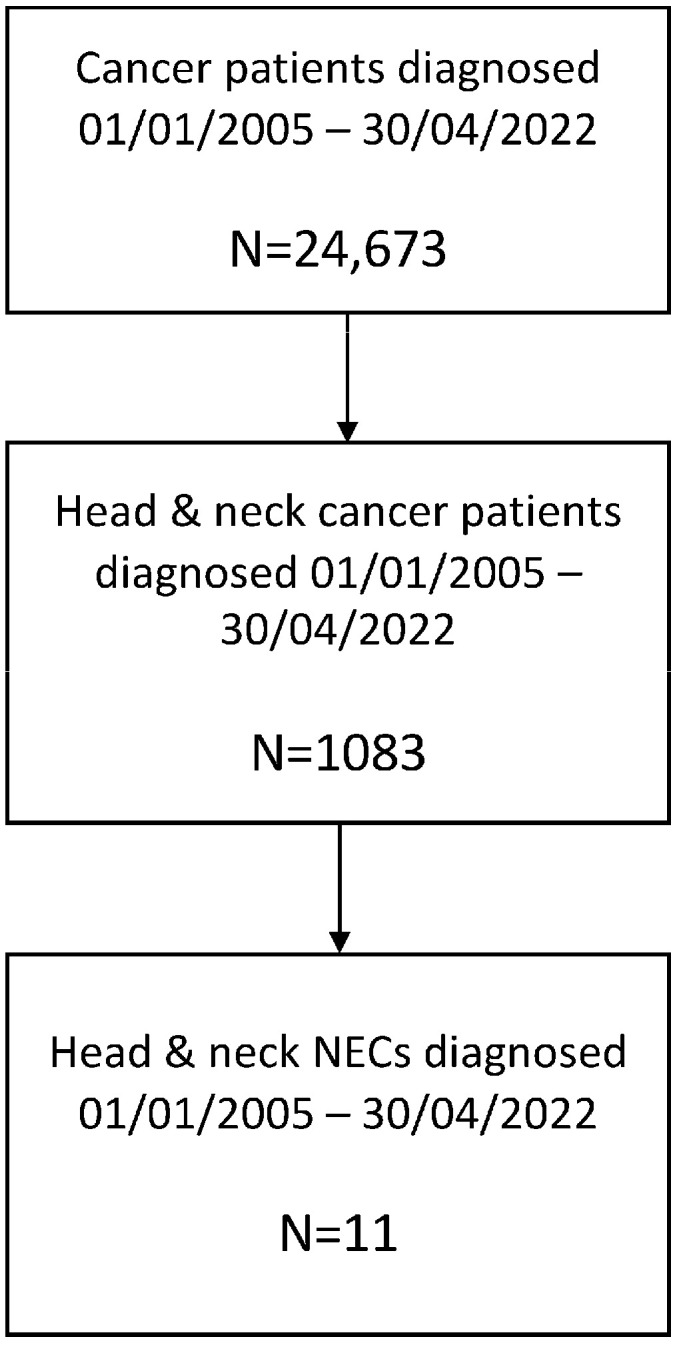
Flowchart showing the patient selection process.

Among 1083 pts diagnosed with HN cancer between January 2005 and April 2022, eleven patients with high-grade HN NECs were identified (Figure 1).

The male: female ratio was 6:5. Median age was 61 (Min–Max: 31–86). Nine of the eleven patients had past or current history of tobacco smoking, and three were current or past heavy alcohol drinkers. HN NEC primary tumors originated from the sinonasal region, major salivary glands, the larynx and the base of the tongue. Ten patients presented with locally advanced or metastatic disease, while only one patient presented with stage II disease. Most cases were of small-cell histology, were poorly differentiated or undifferentiated tumors and, in all cases, harbored a high proliferation index (Ki67 ≥ 40%). Table 1 summarizes the baseline characteristics of the patients, and Table 2 shows the clinical, histopathological and molecular characteristics of each patient.

Among *n* = 8 stage II/IVA/IVB, all received (chemo)radiotherapy with/without prior surgery—three patients were subjected to surgery—and/or prior induction chemo (*n* = 7), with complete response at first evaluation after completing the whole treatment in 7/8 (87.5%).

**Figure 2:**
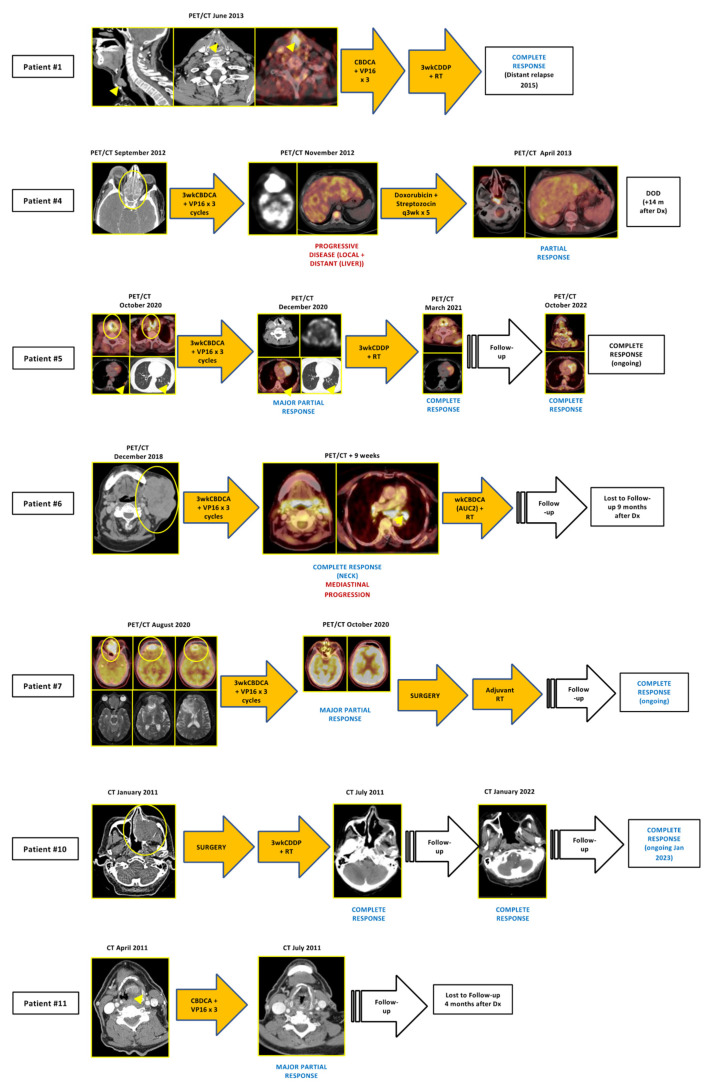
Treatment history of some of the patients with localized HN NECs. For more details, please refer to Table 2. CBDCA: carboplatin, CDDP: cisplatin, CT: computed tomography scan, DOD: dead of disease, PET/CT: 18F-FDG positron emission tomography/computed tomography scan, RT: radiotherapy, VP16: etoposide. Arrowheads and circles indicate tumor lesions.

**Figure 3:**
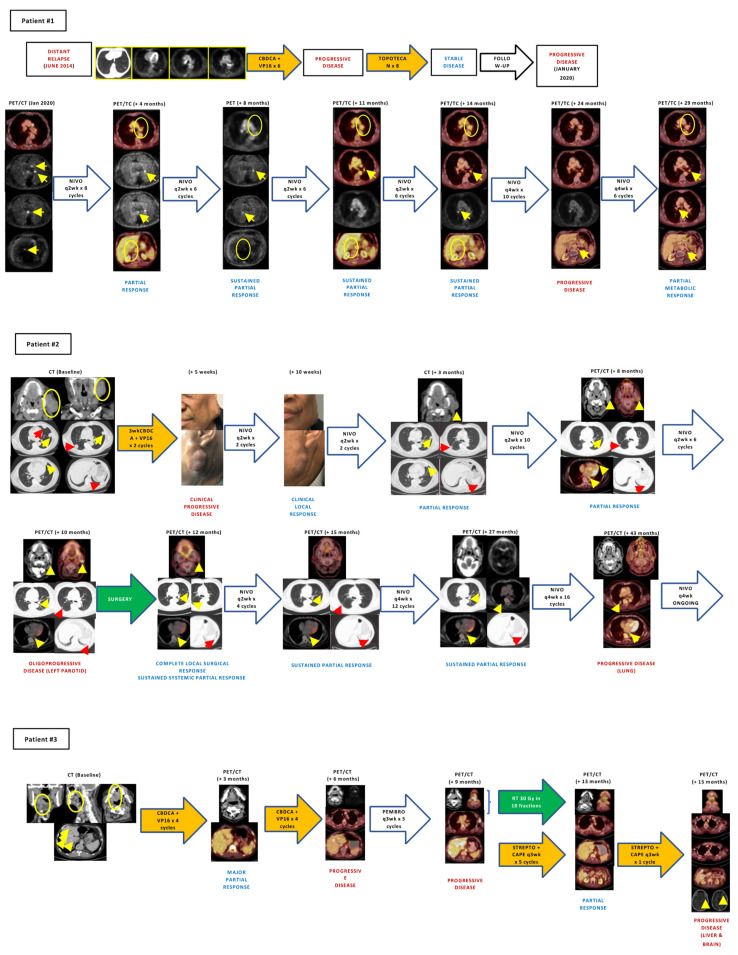
Patients with metastatic HN NECs treated with immune checkpoint inhibitors. **Patient #1:** A 60-year-old man of Afro-Caribbean ethnicity, past smoker of 20 pack-years was diagnosed with a submandibular large-cell neuroendocrine carcinoma in February 2017. Immunohistochemistry was positive for synaptophysin and chromogranin, and the proliferative index as per the Ki67 was 90%. Treatment with three-weekly carboplatin (AUC 5; day 1) plus etoposide (100 mg/m^2^ days 1–3) was started achieving a partial response followed by rescue surgery and adjuvant radiotherapy that ended in March 2018 (66 Gy). Progressive lymph-node disease in left parotid gland occurred in May 2018; an incomplete rescue surgery (superficial lobe parotidectomy) was performed with pathology informing of a poorly differentiated carcinoma with positive margins. Due to remanent macroscopic disease, first-line therapy with cisplatin (50 mg/m^2^), doxorubicin (50 mg/m^2^) and cyclophosphamide (500 mg/m^2^) every 21 days was administered for three cycles followed by 2 cycles without cisplatin due to myelotoxicity, with progressive disease in the left parotid gland and lungs in September 2018. The patient was then transferred to our hospital for a second opinion, and retreatment with two cycles of 3wkCDDP (100 mg/m^2^; day 1) + etoposide (100 mg/m^2^; days 1–3) every three weeks was administered with rapid tumor progression in the left parotid gland and in the bilateral pulmonary metastases. Third-line treatment with nivolumab 240 mg IV every two weeks was started through a compassionate use authorization, achieving a rapid and major partial response in the left parotid and bilateral pulmonary nodules, with excellent tolerance. Only a grade 2 vitiligo in the forehead appeared after one year of therapy and slowly resolved thereafter. Due to progressive disease in the left parotid gland, the patient underwent rescue surgery in September 2019, with pathology informing of a high-grade undifferentiated carcinoma. Treatment with nivolumab was restarted maintaining a complete response in the surgical bed and an asymptomatic progressive disease in a pulmonary lung nodule in the 18F-FDG-PET/CT scan performed in September 2022. **Patient #2:** A 71-year-old man, past smoker of 40 pack-years and past drinker of 100 cc of alcohol per day for 20 years, was diagnosed with a small-cell neuroendocrine carcinoma of the larynx in July 2013. The patient presented a transglottic mass in the left supraglottis that extended bilaterally achieving the subglottis and a right cervical level III lymphadenopathy. Immunohistochemistry was positive for synaptophysin and CD56, being negative for chromogranin, and CK5/6. Proliferative index as per Ki67 was 90%. The patient received three cycles of induction chemotherapy with three-weekly carboplatin (AUC 5; day 1) plus etoposide (100 mg/m^2^ days 1–3) + Filgastrim (300 mcg SC; days 4–10), achieving a partial response in the larynx with progressive disease in the right cervical lymphadenopathy. Treatment with radical chemoradiation with two cycles of three-weekly cisplatin (100 mg/m^2^ days 1 and 22). The third cisplatin cycle was not administered due to grade 3 myelotoxicity. 18F-FDG-PET/CT scan in March 2014 informed of a complete response but suffered progressive disease in the lungs and the right cervical and mediastinal lymph nodes in September 2014. First-line therapy with three-weekly carboplatin (AUC 5; day 1) plus etoposide (100 mg/m^2^ days 1–3) + Filgastrim (300 mcg SC; days 4–10) was started achieving a complete response in the lungs and a partial response in lymph nodes and progressive disease in mediastinal and infradiaphragmatic lymph nodes after 8 cycles in July 2015. Second-line topotecan (2.4 mg IV days 1–5 every 21 days) was started achieving a partial response after three cycles and maintained after 7 cycles when treatment was stopped due to myelotoxicity, and close follow-up ensued. An asymptomatic progression in mediastinal and retroperitoneal lymph nodes occurred in January 2020 when the patient was 78 years old. Treatment with third-line nivolumab 240 mg IV every two weeks was started through a compassionate use authorization, achieving a major partial response after 6 cycles in May 2020 which was still ongoing in the last 18F-FDG-PET/CT scan performed in December 2022. **Patient #3:** A 72-year-old man, past smoker of 45 pack-years, was diagnosed with a metastatic small-cell neuroendocrine carcinoma of base of tongue in June 2021. In addition to the 3 cm ulcerated mass in the base of the tongue, there were a 1.5 cm right paraoesophageal lymphadenopathy, an 8 mm middle-lobe pulmonary nodule and multiple liver occupying lesions, all compatible con metastases. Immunohistochemistry was positive for synaptophysin, CD56 and p16 and negative for chromogranin, p40 and CK20. Proliferative index as per Ki67 was 95%. First-line therapy with three-weekly carboplatin (AUC 5; day 1) plus etoposide (100 mg/m^2^ days 1–3) + Pegfilgastrim (6 mg SC; day 2). After 5 cycles (with 25% reduction since the second cycle due to grade 4 neutropenia in the first cycle), the patient achieved a near-complete radiological response in October 2021. In February 2022, after completing 8 cycles of carboplatin-etoposide, hepatic and lymph-node progression occurred. Treatment with pembrolizumab 200 mg IV every three weeks was started through a compassionate use authorization. After 5 cycles of pembrolizumab, the patient’s condition progressively deteriorated with severe pain requiring opioids in the base of tongue, solid intake dysphagia and asthenia due to tumor progression in the base of the tongue, lymph nodes and liver in the 18F-FDG-PET/CT scan in May 2022. DPYD determination reported no DPYD deficit. Third-line streptozocin (1 g/m^2^; day 1) + capecitabine (625 mg/m^2^/12 h; days 1–14) every 21 days was started with reduced dose of capecitabine due to ECOG 2. After the first cycle, palliative radiotherapy over the relapsed primary tumor in the base of tongue was administered (20 Gy in 5 fractions) with rapid pain relief and no relevant toxicity. Capecitabine dose was progressively increased from 625 mg/m^2^/12 h to 1000 mg/m^2^/12 h due to improved performance status. However, after the fourth cycle, the patient was admitted to the hospital due to grade 4 oral mucositis, grade 4 palmoplantar erythrodysestesia and grade 4 myelotoxicity that slowly resolved during a 4-week in-hospital admission. 18F-FDG-PET/CT scan in September 2022 demonstrated a major partial response. The patient suffered liver progression and developed brain metastases in November 2022 that were treated with stereotactic radiosurgery. Unfortunately, in December 2022, the patient passed away due to respiratory sepsis. Cape: capecitabine, CBDCA: carboplatin, CT: computed tomography scan, DOD: dead of disease, Nivo: nivolumab, Pembro: pembrolizumab, PET/CT: 18F-FDG positron emission tomography/computed tomography scan, RT: radiotherapy, Strepto: streptozotocin, VP16: etoposide. Arrowheads and circles indicate tumor lesions.

Six patients suffered from recurrent and/or metastatic (R/M) diseases, three of them presenting with metastatic disease at initial diagnosis and the other three with recurrent disease, and received a median number of two lines of systemic therapy. Of these, three patients received anti-PD1 agents (nivolumab (*n* = 2), pembrolizumab (*n* = 1)) as the second or third line. Two of them achieved major partial responses, and anti-PD1 therapy is still ongoing after 35 and 49 months, respectively. Figure 2 and Figure 3 depict tumor evolution in each patient.

**Figure 4:**
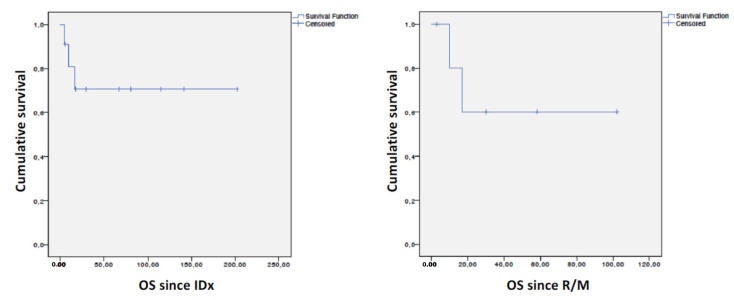
Kaplan–Meier curves of overall survival since initial diagnosis (**left**) and since recurrent/metastatic disease (**right**). IDx: initial diagnosis, R/M: recurrent metastatic disease.

After a median follow-up since initial diagnosis (IDx) of 30 months (Min–max: 5–202) since initial diagnosis (IDx) in the whole cohort (*n* = 11) and of 23.5 months (Min–Max: 3–102) since the R/M setting (*n* = 6), median OS since IDx in the whole series (*n* = 11), as well as since the R/M (*n* = 6) setting, was not reached (Figure 4).

### 3.2. Immunohistochemistry Studies

All patients expressed cytokeratin markers (most commonly CKAE1-AE3). All but two patients expressed synaptophysin (nine positive, one negative, one not available), and four out of ten patients expressed chromogranin. Ki67 was determined in seven patients, six of which showed a Ki67 ≥90% (Table 2).

### 3.3. Tumor Mutational Profile and Tumor Mutational Burden (TMB)

The median TMB (*n* = 7 patients) was 6.72 Muts/Mb (Min–max: 0.84–15.94). The most common pathogenic gene variants occurred in TP53 and HNF1A, with other pathogenic variants detected in SMARCB1, CDKN2A, PIK3CA, RB1 and MYC. Table 3 and Supplementary Table S1 summarize the TMB and pathogenic gene variants detected in each of the cases analyzed.

### 3.4. Clonal T-Cell Receptor Repertoire

The median number of TCR clones in *n* = 5 patients analyzed was 224 (Min-max: 59–1446). In patient #2 (see Figure 3), the number of TCR clones increased from 59 to 1446 after nivolumab treatment. Table 3 and Supplementary Table S2 show the TCR repertoire among all the cases analyzed.

### 3.5. Survival and Molecular Profile

There were no statistically significant survival differences since IDx and since the R/M setting based on TP53 status (mutated vs. wild-type), TMB value and the number of pathogenic mutations detected. Likewise, there was no statistically significant correlation between survival since IDx and since the R/M setting and TMB value and TCR clonality. These results are summarized in Table 4. In addition, there were no statistically significant associations between clinical characteristics and survival since IDx and since the R/M setting (Supplementary Table S3).

## 4. Discussion

HN NECs are extremely rare tumors, with the largest series reporting a 1% prevalence among HN cancers (3)(4)(7). We identified 11 patients with HN NECs among 1083 diagnosed with HNC of any histology in an 18-year period, thereby accounting for 0.93% of HN cancers in our series. As expected, most patients were male and past or current tobacco smokers, in agreement with the well-known association between tobacco and NECs of the HN region [1,3,4,7,12,13,14,15,16,17,18,19,20,21,22]. Most tumors were either small-cell or mixed-cell carcinomas, harbored a very high proliferative index (>90%), expressed prototypical neuroendocrine markers such as synaptophysin and/or chromogranin and presented with locally advanced or metastatic disease, developing, in descending order, from major and minor salivary glands, sinonasal structures and the larynx, all these concordant with prior reports (Table 5 summarizes the most relevant studies conducted to date in HN NECs) [1,3,4,7,12,13,14,15,16,17,18,19,20,21,22].

As shown in Figure 4, while median OS since IDx and since the R/M setting were not reached, some patients died early, possibly reflecting late diagnosis or biologically more aggressive diseases. However, we found no clinical or biological differences in OS, although this may be explained by the small sample size of our series.

Among the eight patients diagnosed with localized disease (stages II-IVA/B), treatment consisted in platinum-based chemoradiotherapy (CRT) with or without induction chemotherapy with PE. Only two patients underwent surgery as part of their multimodality treatment. OS in these eight patients was durable despite three patients relapsing after therapy. Contrary to prior studies, where induction chemotherapy has been seldom used and where most patients underwent either upfront surgery followed by (C)RT or upfront CRT, in our series, we show that multimodality organ-preservation approaches using induction PE-based chemotherapy followed by platinum-based CRT constitute a successful therapeutic strategy for HN NECs [4,7,14,15,16,17,19]. Similar organ-preservation strategies are widely used in squamous cell carcinomas of the head and neck, particularly in patients with laryngeal cancer [23]. Interestingly, in two of the patients in our series diagnosed with laryngeal NECs, this multimodality approach achieved complete locoregional responses with induction PE-based therapy followed by concomitant cisplatin-based CRT. However, NECs developing in sinonasal structures may preferentially be managed with surgery with or without prior neoadjuvant PE-based chemotherapy given the frequent invasion of bone structures where it might be more cumbersome for both chemotherapy and radiotherapy to penetrate [1,4,17,18].

Of note, among the six patients with metastatic disease, median OS was not reached after a median follow-up of 23.5 months. This may reflect biological differences compared to NECs outside the HN region, such as SCLC, which are known to harbor 12-month OS or less, as well as therapeutic advances such as the advent of anti-PD1 agents, the latter used in three of our patients [24,25,26]. One patient with a locally advanced disease and a single 1 cm lung metastasis at initial presentation, achieved a complete response with induction chemotherapy still ongoing 27 months after therapy. Two patients with d’emblée metastatic disease lived for 14 and 18 months, respectively, the latter of them treated with pembrolizumab after platinum-based therapy. In addition, two other patients with metastatic lymphadenopathies with or without pulmonary metastases received the anti-PD1 nivolumab achieving major and durable responses, with both patients still on treatment, 35 and 49 months after the start of immunotherapy. This is notable, since, as far as we know, this is the first article reporting on HN NEC patients treated with immune checkpoint inhibitors to date and demonstrating that anti-PD1 agents may achieve profound and durable responses in heavily platinum-pretreated patients with NECs of the HN region. The largest evidence to date on the use of immune checkpoint inhibitors (ICIs) in NECs comes from extensive-stage small-cell lung cancer (ES-SCLC). In the CheckMate 032 trial, ORR and median OS with nivolumab were 11.6% and 5.7 months, respectively, and with nivolumab plus ipilimumab, they achieved 21.9% and 4.7 months, respectively [27]. First-line atezolizumab plus PE was approved in ES-SCLC after demonstrating a median OS of 12.3 months compared to 10.3 months for the placebo plus chemotherapy group (HR 0.73; 95% ci, 0.60 to 0.95; *p* = 0.0154) [23,24]. In the Keynote-604 study, pembrolizumab-PE increased progression-free survival (PFS) and numerically increased OS compared to placebo-PE in ES-SCLC [25]. In the CASPIAN study, durvalumab-PE or durvalumab-tremelimumab-PE increased OS (HR 0.71, 95% CI 0.60–0.86, *p* = 0.0003; HR 0.81, 95% CI 0.67–0.97, *p* = 0.0200) over placebo-PE [28]. In the ECOG-ACRIN EA5161 study, the combination of nivolumab-PE significantly increased PFS and OS compared to placebo-PE in ES-SCLC [29]. Finally, two systematic reviews and meta-analyses concluded that chemo-immunotherapy combinations significantly increase OS and PFS in ES-SCLC [30,31].

In addition, small, non-randomized studies with immune checkpoint inhibitors have been conducted in extra-thoracic high-grade neuroendocrine tumors. Among 29 patients with GEP-NETs participating in two phase II, open-label, second-line trials that received pembrolizumab, disease control rate was 24.1% with only one partial response [32]. In a seven-patient phase II study of female patients with small-cell NECs of the lower genital tract, treated with pembrolizumab, there was one disease stabilization and six progressions [6].

Little is known about the immune profile of HN NECs [16,17]. We could only determine the PD-L1 status in tumor cells in two patients from our series, one of them with a TPS = 0 and the other with a TPS = 5%, and both received nivolumab in the fourth and third line, respectively, achieving very durable benefit which is still ongoing. Strojan et al. [16] evaluated the PD-L1 status reporting a CPS ≥ 1 in 2/19 patients. Despite the limited evidence, these results suggest that PDL1 expression might not be common in HN NECs and may not predict the benefit from ICIs in this disease, in agreement with what has been shown in SCLC [33]. In five of the patients in our series, TCR clonality was analyzed, demonstrating a high number of clones with >100 T-cell clones in four of the patients. Interestingly, in one of these patients, subjected to surgery for oligoprogressive disease, the number of TCR clones increased from 59 to 1446 after nivolumab treatment. Anti-PD1 agents administered as neoadjuvant therapy have been shown to increase T-cell clonality in non-small cell lung cancer, through an increase in anti-tumor T-cell quantity and diversity [11,34]. To our knowledge, this is the first case showing an increase in T-cell clonality after treatment with immunotherapy in an NEC of any anatomical origin.

In our study, median TMB was 6.72 mut/Mb (Min-max: 0.84–15.94), which is similar to the findings of a recent study by Ohmoto et al. [17], reporting a median TMB of 7.1 mut/Mb (range 3.9–17.2). Among the seven patients analyzed in our study, two patients showed a TMB ≥13 muts/Mb. The patient with the lowest TMB (0.84) was an 87-year-old female with no history of tobacco or alcohol exposure, indicating that HN NECs may also develop in patients devoid of typical risk factors. In our series, we found no association nor correlation between survival and TMB. Though a high TMB (≥10 muts/Mb) has been claimed to predict benefit from anti-PD1 agents, reports have been controversial, with different results depending on the type of cancer and methodological aspects such as the type of TMB test used and cut-off point established [32,35,36,37]. In June 2020, the FDA made an agnostic approval for pembrolizumab for patients with any solid tumor and a TMB ≥ 10 muts/Mb [38]. As shown in our series, two patients showed a TMB above 10 muts/Mb. Among the three patients treated with anti-PD1 agents, TMB was determined in two of them, achieving 6.71 and 6.72 muts/Mb, respectively. The first of them responded to fourth-line nivolumab while the second progressed to second-line pembrolizumab, thus indicating that TMB alone may be insufficient to predict benefit from immunotherapy in HN NECs [39]. In a retrospective cohort study of solid tumors not treated with immunotherapy, those most commonly harboring a high TMB, defined as a TMB ≥ 10 muts/Mb, were SCLC (40%) and NETs (29.3%) [40]. In the IMPower133 study in ES-SCLC patients treated with first-line atezolizumab-PE derived benefit regardless of PD-L1 expression or blood TMB (bTMB), although patients with a bTMB ≥ 16 muts/Mb had a longer OS in the atezolizumab group compared to those with a bTMB < 16 muts/Mb [24]. In the CheckMate-032 study, patients with a TMB in the highest tertile showed a higher ORR with nivolumab +/− ipilimumab, a longer PFS with nivolumab + ipilimumab and a longer OS with nivolumab +/− ipilimumab [33]. Therefore, while in ES-SCLC TMB seems to be predictive of benefit from ICIs, it remains to be demonstrated in HN NECs.

Mismatch-repair deficiency (MMRd) has been shown to predict benefit from immune checkpoint inhibitors in colorectal, endometrial and germline-MMRd cancers [41]. Evidence on the existence of MMRd in head and neck cancers is very limited. Hieggelke et al. [42], in a recent study, detected 3.2% of sinonasal squamous cell carcinoma with MMRd. To our knowledge, there are no reports on the prevalence of MMRd in NECs of the HN region. Unfortunately, we could not analyze the MMRd status in our patients’ tumors.

In our series, the study of the mutational profile revealed pathogenic gene variants in TP53, RB1 and MYC, which are characteristic of SCLC and closely linked to tobacco smoking [43]. In the study by Ohmoto et al. [17], the most frequent mutations also occurred in TP53 and RB1, followed by mutations in NOTCH1, PIK3CA and CDKN2A, among others. Peng et al. [18] also reported mutations in TP53 and RB1, in addition to mutations in NOTCH2 and PTEN, among others. Moreover, in the study by Ohmoto et al. [17], actionable fusions were reported, such as the FGFR3-TACC3 fusion in one patient and the SEC11C-MYC fusion in another patient. In our study, we found no association nor correlation between survival and TP53 status and the number of pathogenic mutations, although any conclusion in this regard is limited because of the few patients included.

Recently, a new methylation-based classification for sinonasal tumors has been proposed, recognizing four molecular subtypes and sinonasal undifferentiated tumors (SNUC), two of them with NEC-like features (NEC-like IDH2 and NEC-like SMARCA4/ARID1A), a third group characterized by SMARB1 loss and a fourth one with characteristics of adenoid cystic carcinoma [44]. In our series, four patients harbored sinonasal NECs that expressed prototypical neuroendocrine markers such as synaptophysin and chromogranin and showed very high proliferative indexes. In one of the two tumors analyzed through NGS in our study, mutations in ARID1A and SMARCB1 were detected. This patient is disease-free 28 months after diagnosis, which would agree with the NEC-like SMARCA4/ARID1A enriched group of SNUC tumors, which are believed to derive from diffuse neuroendocrine cells and harbor a more favorable prognosis with a 5-year OS of 68% [44].

Our study is limited by its retrospective nature and small sample size. While all of our patients received multimodality treatment in the early and locally advanced settings, the optimal approach and, in particular, the roles of induction chemotherapy and the indication for surgery instead of organ-preservation approaches are yet to be precisely defined. We could not determine the PDL1 status in most patients or study immune cell subpopulations or the expression of immune markers known to play a role in innate and acquired immunity, thereby limiting the findings about the immune profile of the tumor. Finally, only three patients in our series received immune checkpoint inhibitors. Therefore, the conclusions on their efficacy and safety in this entity cannot be generalized until larger prospective studies are conducted.

## 5. Conclusions

HN NECs are biologically aggressive but may achieve long-lasting survival with multimodality treatment; despite this, other patients will succumb early to their disease. In our series, NECs showed moderate-high TMBs and large TCR repertoires, which may explain responses to anti-PD1 agents in some patients and justify the further study of immunotherapy in this disease.

## Figures and Tables

**Table 1:** Summary of demographic and clinicopathologic characteristics of the patients.

Characteristics	Patients (*n* = 11)
Age in years at IDx, median (Min–Max) (*n* = 11)	61 (30–87)
Age in years at R/M, median (Min–Max) (*n* = 7)	68 (50–87)
Sex, *n* (%)	
Male	6 (54.5%)
Female	5 (45.5%)
Ethnicity, *n* (%)	
Caucasian	10 (91%)
Afro-Caribbean	1 (9%)
Smoking history, *n* (%)	9 (82%)
Primary tumor, *n* (%)	
Sinonasal	3 (27%)
Parotid gland	3 (27%)
Submaxillary gland	1 (9%)
Larynx	3 (27%)
Base of tongue	1 (9%)
Stage at initial diagnosis (AJCC), *n* (%)	
II	1 (9%)
IVA	3 (27.3%)
IVB	4 (36.4%)
IVC	3 (27.3%)
Histology, *n* (%)	
Small cell	7 (64%)
Mixed small/large cells	4 (36%)
Poorly/undifferentiated	10 (91%)
Ki67, median (Min–Max)	90 (40–100)

AJCC: American Joint Committee on Cancer, IDx: initial diagnosis, *n*: number of patients, R/M: recurrent/metastatic disease.

**Table 2:** Disease characteristics of each of the patients included in the current study.

Patient No.	Age, Stage and Treatment at IDx	Immunohistochemical Features	Molecular Profile	Age and Treatment at R/M Disease	PFS and OS Since IDx	PFS and OS Since R/M Disease/PFS and OS Since Start of IO
#1	71 y/Laryngeal small-cell stage II NEC/Smoker, heavy drinker/CBDCA (AUC5) + VP16 × 3 → RT + 3wkCDDP (100 mg/m^2^) × 2	CKAE1-AE3 (+), CK5/6 (−), Chromogranin (−), Synaptophysin (+), CD56 (+). Ki67 = 90% PD-L1 (TPS): 5%	TCR (pre-Nivo): 211 clones	72 y/CBDCA (AUC5) + VP16 × 6 → PD → Topotecan × 8 → SD → W&S (4 years) → PD → Nivo 240 mg q2wk × 6 m → PR → Nivo 480 mg q4wk × 30 m → PD → Nivo BPD (ongoing)	PFS: 9 m OS: 115 m	PFS R/M: 6 m OS R/M: 103 m PFS IO: 24 m OS IO: 35 m
#2	59 y/Submandibular gland poorly differentiated stage IVA NEC/Non-smoker /CBDCA (AUC5) + VP16 × 3 → PR → SX → RT + Cetuximab → PD → SX (partial resection)	CKAE1-AE3 (+), CK5/6 (−), Chromogranin (-), Synaptophysin (+), CD56 (−). Ki67 NA. PD-L1 (TPS): 0	TMB (pre-Nivo): 6.72 mut/Mb TMB (post-Nivo): 5.02 mut/Mb TCR (pre-Nivo): 59 clones TCR (post-Nivo): 1446 clones	60 y/CAP × 3 → PD → DI × 2 → PD → CBDCA (AUC5) + VP16 × 2 → PD → Nivo 240 mg q2wk × 2 m → PR → Nivo 240 mg q2wk × 8 m → PD → SX (oligomtx) → Nivo 240 mg q2wk × 19 m → Nivo 480 mg q4wk × 18 m → PD → Nivo BPD (ongoing)	PFS: 14 m OS: 71 m	PFS R/M: 3 m OS R/M: 54 m PFS IO: 10 m OS IO: 49 m
#3	71 y/Minor salivary gland base of tongue small-cell stage IVC NEC/Smoker /CBDCA (AUC5) + VP16 × 4 → PR → CBDCA (AUC5) + VP16 × 4 → Pembro 200 mg/q3wk × 5 → PD → Stretozocin 1 g + Capecitabine 800 mg/m^2^/bid q3wk × 3 → PR → Stretozocin + Capecitabine × 1 → PD	CKAE1-AE3 (+), CK5/6 (−), Chromogranin (−), Synaptophysin (+), CD56 (+). Ki67 = 95%	Mut: TP53 TMB: 6.71 mut/Mb	(See 2nd column)	PFS: 6 m OS: 18 m	PFS R/M: 6 m OS R/M: 18 m PFS IO: 3 m OS IO: 8 m
#4	56 y/Nasoethmoidal small-cell stage IVC NEC/Smoker, heavy drinker/CBDCA (AUC5) + VP16 × 4 → PD → Stretozocin + Doxorubicin q3wk × 5 → PR → PD	CKAE1-AE3 (+), Chromogranin (+), Synaptophysin (+), NSE (+), CD99 (−). Ki67 = 50%	Mut: CDKN2A, TP53 TMB: 15.94 mut/Mb	(See 2nd column)	-	PFS R/M: 3 m OS R/M: 14 m
#5	68 y/Laryngeal mixed small- and large-cell stage IVC NEC/Smoker /CBDCA (AUC5) + VP16 × 3 → RT + 3wkCDDP (100 mg/m^2^) × 3 → CR (ongoing)	CKAE1-AE3 (+), Chromogranin (−), Synaptophysin (+), Ki67 = 100%	Mut: RB1, TP53 TMB: 11.83 mut/Mb TCR: 4025 clones	(See 2nd column)	-	PFS R/M: 27 m OS R/M: 27 m
#6	86 y/Parotid small-cell stage IVB NEC/Non-smoker, non-drinker/CBDCA (AUC4) + VP16 × 3 → RT	CKAE1-AE3 (+), Chromogranin (−), Synaptophysin (+), CD56 (+). Ki67 = 90%	Mut: PIK3CA, HNF1A TMB: 0.86 mut/Mb	-	PFS: 10 m OS: 10 m	PFS R/M 1 m OS R/M: 1 m (lost to FU afterward)
#7	56 y/Nasoethmoidal large-cell stage IVB NEC/Smoker /CBDCA (AUC5) + VP16 × 3 → PR → SX → RT → CR (ongoing)	CKAE1-AE3 (+), Chromogranin (+), Synaptophysin (+), CD99 (+). Ki67 NA	Mut: ARID1A, SMARCB1 TMB: 5.05 mut/Mb TCR: 224 clones	-	PFS: 28 m OS: 28 m	-
#8	30 y/Parotid small-cell stage IVA NEC/Smoker, non-drinker/3wkCDDP + VP16 × 2 → CBDCA (AUC5) + VP16 × 1 → RT + CBDCA (AUC5) × 3 → CR (ongoing)	CKAE1-AE3 (+), Chromogranin NA, Synaptophysin NA, CD56 NA, Ki67 NA	-	-	PFS: 78 m OS: 78 m	-
#9	50 y/Submaxillary gland small-cell stage IVA NEC/Smoker, non-drinker/CBDCA (AUC5) + VP16 × 3 → RT + CBDCA (AUC5) × 3 → CR (ongoing)	Cytokeratin (+), Chromogranin (−), Synaptophysin (+), NSE (+), CD56 NA, Ki67 NA	Mut: MYC, HNF1A TMB: 5.06 mut/Mb TCR: 103 clones	-	PFS: 204 m OS: 204 m	-
#10	60 y/Sinonasal NEC stage IVB/Smoker/SX → RT + 3wkCDDP (100 mg/m^2^) × 3 → CR (ongoing)	CKAE1-AE3 (+), Chromogranin (+), Synaptophysin (+), NSE (+), CD99 (+). Ki67 = 90%	-	-	PFS: 132 m OS: 132 m	-
#11	78 y/Sinonasal NEC stage IVB/Smoker/CBDCA (AUC5) + VP16 × 3 → RT + wkCBDCA (AUC2) × 7 → PR	Chromogranin (+), Synaptophysin (−), Ki67 = 90%	-	-	PFS: 5 m OS: 5 m	-

AUC: area under the concentration curve, BPD: beyond progressive disease, CBDCA: carboplatin, CDDP: cisplatin, CR: complete response, DI: doxorubicin plus iphosphamide, IDx: initial diagnosis, IO: immunotherapy, m: months, Mut: mutations, mut/Mb: mutations per megabase, NA: not available, NEC: neuroendocrine carcinoma, Nivo: nivolumab, OS: overall survival, PD: progressive disease, Pembro: pembrolizumab, PFS: progression-free survival, PR: partial response, R/M: recurrent/metastatic disease, SD: stable disease, SX: surgery, TCR: T-cell receptor repertoire, TMB: tumor mutational burden, VP16: etoposide.

**Table 3:** Molecular profile, TMB and TCR repertoire of the tumors analyzed.

	Somatic Pathogenic Mutations		TMB (Mut/Mb)	No. of Clones	Shannon Diversity	Evenness
Patient #1	-	-	-	211	5935	0.769
Patient #2	-	Pre-Nivo	6.72	59	4186	0.712
		Post-Nivo	5.02	1446	5508	0.525
Patient #3	TP53	-	6.71	-	-	-
Patient #4	CDKN2A, TP53	-	15.94	-	-	-
Patient #5	RB1, TP53	-	13.41	4025	10,684	0.892
Patient #6	PIK3CA, HNF1A	-	0.84	-	-	-
Patient #7	ARID1A, SMARCB1	-	5.02	224	6556	0.839
Patient #9	MYC, HNF1A	-	3.35	103	6029	0.902

Nivo: nivolumab, TCR: T-cell receptor repertoire, TMB: tumor mutational burden. (-): not applicable or not available.

**Table 4:** Survival and association and correlation with molecular profile, TMB and TCR repertoire.

	Association			Correlation	
	OS Since IDx	OS Since R/M		OS Since IDx	OS Since R/M
TP53 status (Mut vs. WT)	*p* = 0.221	*p* = 0.157	TCR	r = 0.800 (*p* = 0.104)	r = 0.800 (*p* = 0.200)
TMB (≥6 vs. <6 muts/Mb)	*p* = 0.695	*p* = 0.515	TMB	r = 0.224 (*p* = 0.629)	r = 0.837 (*p* = 0.077)
Mut No. (≥2 vs. <2)	*p* = 0.450	*p* = 0.083	-	-	-

IDx: initial diagnosis, Mb: megabase, Mut: mutant, muts: mutations, *p* = *p*-value, R/M: recurrent/metastatic, TCR: T-cell repertoire, TMB: tumor mutational burden, WT: wild type.

**Table 5:** Summary of most relevant studies published to date on neuroendocrine carcinomas of the head and neck region.

Author (Year)	N (Incidence among HNCs)	H&N Site	Pathology and Molecular Profile	Treatment	PFS/DFS	OS
Ferlito (1986) [12]	14 (Retrospective series 1966–1984)	Larynx	-	SX: 6/14 RT: 12/14 CT: 8/14	-	-
Barker (2003) [19]	23 (Retrospective series 1984–2001)	Larynx: 13/23 OP: 3/23 OC: 1/23 HP: 2/23 NP: 2/23 SG: 2/23	-	RT: 14/23 SX +/− RT: 9/23 CT: 14/23	2 y-DFS: 41% 5 y-DFS: 25%	2 y-OS: 53% 5 y-OS: 33% CT associated with better OS and DMFS
Rosenthal (2004) [20]	72 (Retrospective series 1982–2002)	NC and PNS: 72/72 -ENB: 31/72 -SNUC: 16/72 -NEC: 18/72 -SCNEC: 7/72	-	ENB: -CT: 5/31 (16%) -Local Tx only (RT or SX): 26/31 (84%) Non-ENB: -CT: 27/45 (60%) -RT: 15/45 (33%)	-	ENB: 5 y-OS: 93.1% Non-ENB: 5 y-OS (SNUC): 62.5% 5 y-OS (NEC): 64.2% 5 y-OS (SCNEC): 28.6%
Hatoum (2009) [21]	12 (Retrospective series 1987–2007)	NP: 2/12 NC and PNS: 2/12 SG: 4/12 OP: 3/12 Larynx: 1/12	SCNEC: 12/12	CRT: 7/12 SX/CRT: 2/12 CT: 1/12 RT: 2/12	-	1 y-OS: 71% 2 y-OS: 44%
Kao (2012) [13]	23 → 14 SC/LC	OP: 2/23 Larynx: 9/23 NC and PNS: 11/23 SG: 1/23	LCNEC/SCNEC: 13/14 were P53(+) WD/MD TNEs: 9/9 were P53(−)	-	-	LCNEC/SCNEC: 25.5 m
Servato (2013) [14]	44 (Retrospective review + 2 case reports < 9	All SG NECs (Parotid 35, submaxillary 9)	Chro (+): 29/44 Syn (+): 19/44 CD56 (+): 7/44 NSE (+): 36/44	SX: 38/44 RT: 28/44 CT: 13/44 No: 1/44	-	2 y-OS: 56.4% 5 y-OS: 36.6%
Alos (2016) [1]	19	OP: 1/19 Larynx: 7/19 NC and PNS: 5/19 SG: 6/19	CKAE1/AE3 (+): 19/19 Chro (+): 15/19 Syn (+): 17/19 CD56 (+): 18/19 Ki67 (+): 19/19 HPV DNA (+): 0/19	SX: 17/19 RT: 13/19 CT: 5/19	-	Stage I/II longer OS than III/IV No differences in OS between URT vs. SG
Zhan (2016) [7]	344 (Retrospective review NCDB 1998–2012)	Parotid gland SCNEC	-	SX: 61.9% RT: 64.8% CT: 55.5%	-	5 y-OS: 37% 10 y-OS: 20% Tumor size and distant metastases were prognostic factors for OS
Thomson (2016) [22]	10	OP: 4/10 Larynx: 3/10 NC and PNS: 3/10	Chro (+): 3/10 Syn (+): 9/10 CD56 (+): 5/8 HPV DNA: 3/7	SX: 5/8 RT: 6/8 CT: 7/8 BSC: 1/8	-	-
Pointer (2017) [3]	1042 (Retrospective review NCDB)	OC: 9% OP: 12% Larynx: 35% HP: 4% NP: 10% NC and PNS: 30%	-	-	-	OC: 20.8 m OP: 23.7 m Larynx/HP: 17.9 m NP: 15.1 m NC and PNS: 36.4 m No difference in OS depending on TX for stage I-II No difference in OS in stage III_IVA/B between SX + CRT vs. CRT alone
Wakasaki (2019) [15]	21	Larynx: 6/21 NC and PNS: 5/21 HP: 3/21 OP: 2/21 NP: 2/21 OC: 1/21 UP: 1/21 SG: 1/21	-	SX: 9/21 RT: 5/21 CRT: 7/21 CT: 6/21	-	1 y-OS: 56% 3 y-OS: 37%
Issa (2021) [4]	415	NC: 52.5%	-	RT: 30% CRT: 27.2% SX/CRT: 11.6%	-	Trimodal and bimodal TX better OS vs. unimodal TX CRT or SX/CRT increased OS
Strojan (2021) [16]	20	OP: 2/20 Larynx: 12/20 HP: 3/20 NP: 3/20	WD TNE: 1/20 MD TNE: 4/20 PD SC/LC: 15/20 CKAE1/AE3 (+): 19/19 Chro (+): 6/20 Syn (+): 3/20 CD56 (+): 17/17 Ki67 (+): 20/20 HPV DNA (+): 2/20 CPS (PDL1) > 1: 2/19	SX/RT/CT: 1/20 SX/RT: 4/20 SX/CT: NA RT/CT: 8/20 SX: 5/20 CT: NA BSC: NA	-	2 y-OS: 64% 5 y-OS: 34%
Ohmoto (2021) [17]	27	OC: 4% OP: 19% Larynx: 7% HP: 11% NC and PNS: 48% SG: 11%	SCNEC: 10/24 LCNEC: 14/24 Mutations (*n* = 14): TP53: 6/14 RB1: 3/14 NOTCH1: 2/14 PIK3CA: 1/14 FAT1: 1/14 CDKN2A: 1/14 SMARC4: 1/14 HIST3H3: 1/14 PREX2: 1/14 Fusions (*n* = 14): FGFR3-TACC3: 1/14 SEC11C-MYC: 1/14 TMB (*n* = 14): 7.1 mut/Mb (range, 3.9–17.2).	SX/RT/CT: 15% SX/RT: 7% SX/CT: 4% RT/CT: 22% SX: 15% CT: 11% BSC: 7%	3-y RFS (*n* = 17 LAD): 27%	3-y OS (*n* = 27): 39% 3-y OS (*n* = 17 LAD): 53%
Peng (2021) [18]	5 with 2^nd^ primary HNC after NPC	OC: 1/5 NC and PNS: 4/5	Chro (+): 5/5 Syn (+): 5/5 CD56 (+): 5/5 EBER (+): 0/5 Mutations (*n*=2/5): TP53, NOTCH2, PTEN, RB1, POLG, KMTWC, U2AF1, EPPK1, ELAC2, DAXX, COL22A1, ABL1	SX: 4/5 RT: 3/5 CT: 5/5	PFS: 3–6 m	OS: 3–9 m
Current series	11 (Retrospective series 2005–2022)	NC and PNS: 3/11 SG: 4/11 OP: 1/11 Larynx: 3/11	SCNEC: 7/11 MCNEC: 4/11 PD-L1 (TPS): 0 and 5% in 2 pts tested. Mutations (*n* = 7): RB1, TP53, CDKN2A, PIK3CA, ARID1A, SMARCB1, HNF1 TMB (*n* = 5): 6.72 Muts/Mb (Min-max: 0.84–15.94).	RT: 30% CRT: 27.2% SX/CRT: 11.6%	-	OS (IDx) (*n* = 11): NR OS (R/M) (*n* = 6): NR

BSC: best supportive care, Chro: chromogranin, CPS: combined positive score, CT: chemotherapy, DFS: disease-free survival, ENB: esthesioneuroblastoma, HNC: head and neck cancer, H&N: head and neck, HP: hypopharynx, LC: large cell, MCNEC: mixed-cell NEC, MD: medium-sized cell, MD: moderately differentiated, Muts/Mb: mutations per megabase, NC: nasal cavity, NCDB: national cancer database, NECs: neuroendocrine carcinomas, NETs: neuroendocrine tumors, NP: nasopharynx, NPC: nasopharyngeal carcinoma, NSE: neuronal specific enolase, OC: oral cavity, OP: oropharynx, OS: overall survival, PNS: paranasal sinuses, RT: radiotherapy, SC: small cell, SG: salivary gland, SX: surgery, Syn: synaptophysin, TMB: tumor mutational burden, TPS: tumor proportion score, TX: therapy, UP: unknown primary, WD: well differentiated.

## Data Availability

The datasets used and/or analyzed during the current study are available from the corresponding author on reasonable request.

## Supplementary Materials

The following supporting information can be downloaded at https://www.mdpi.com/article/10.3390/cancers15092431/s1, Table S1: Supplementary Material Pathogenic gene variants detected in each of the analyzed cases using NGS, Table S2: Supplementary Material TCR repertoire analyzed through NGS. Table S3. Association of survival with clinical characteristics.
