# Supplementary material for: Clinical Behavior, Mutational Profile and T-Cell Repertoire of High-Grade Neuroendocrine Tumors of the Head and Neck
Source: Cancers (Basel). 2023 Apr 24;15(9):2431. doi: 10.3390/cancers15092431 (PMC10177201; doi:10.3390/cancers15092431)
Supplement: Supplementary file 1 [file cancers-15-02431-s001.zip › cancers-2316207-supplementary/Supplementary Table S3.pdf]

Supplementary Table S3. Association of survival with clinical characteristics.

	Association	
	**OS since IDx**	**OS since R/M**
**Sex (Male vs Female)**	P=0.105	P=0.445
**Smoking (Yes vs No)**	P=0.319	P=0.445
**Primary tumor (PNS vs SG vs OP vs Larynx)**	P=0.463	P=0.129
**Stage IDx (II vs IVA vs IVB vs IVC)**	P=0.387	P=0.438
**Ki67 (< 90 vs ≥90)**	P=0.356	P=0.246
**Chromogranin (positive vs negative)**	P=0.193	P=0.808
**Synaptophysin (positive vs negative)**	P=0.157	P=0.445
**Neuronal specific enolase**	P=0.480	P=0.317

IDx: initial diagnosis, P= p-value, PNS: paranasal sinus, R/M: recurrent/metastatic, SG: salivary gland, OP: oropharynx
